# Left Atrial Appendage Occlusion on a Beating Heart during Minimally Invasive Valve Surgery Using an Aortic Endoclamp: A Case Report

**DOI:** 10.3390/jcm12196325

**Published:** 2023-09-30

**Authors:** Mathieu N. Suleiman, Ann-Sophie Kaemmerer, Jörg Fechner, Ehab Nooh, Michael Weyand, Christian Heim

**Affiliations:** 1 Department of Cardiac Surgery, University Hospital Erlangen, Friedrich-Alexander-University Erlangen-Nürnberg, 91054 Erlangen, Germany; askaemmerer@gmail.com (A.-S.K.); ehab.nooh@uk-erlangen.de (E.N.); michael.weyand@uk-erlangen.de (M.W.); christian.heim@uk-erlangen.de (C.H.); 2 Department of Anesthesiology, University Hospital Erlangen, Friedrich-Alexander-University Erlangen-Nürnberg, 91054 Erlangen, Germany; joerg.fechner@uk-erlangen.de

**Keywords:** mitral valve prolapse, minimally invasive surgery, mitral valve replacement, IntraClude Device, AtriClip

## Abstract

Concomitant LAA occlusion has been shown to be an effective and safe treatment for patients with atrial fibrillation during cardiac surgery to prevent embolic stroke. Minimally invasive procedures are challenging due to restricted access to and visibility of the surgical site. Also, aortic endoclamping has been developed as an alternative surgical approach to exoclamping. The aim of this article is to demonstrate the method of beating heart LAA occlusion with the Atriclip^®^ (AtriCure, Mason, OH, USA) device during minimally invasive mitral valve surgery while using the endoclamping alternative for aortic cross-clamping.

## 1. Introduction

With the progress in cardiac surgery toward minimally invasive procedures, new challenges have arisen. The limited exploration of the surgical field, limited visibility in terms of optical angulations and longer distances contribute to a steep learning curve [1,2].

Atrial fibrillation (AF) is common in patients with heart disease and, if left untreated, is associated with a high risk of thromboembolic events. Oral anticoagulation plays an important role in preventing AF-related thromboembolic complications [3]. Today, complex interventional or surgical options for the treatment of AF and the prophylaxis of thromboembolism are also available.

The prevalence of AF in patients with mitral valve regurgitation and atrial dilatation is high. The left atrial appendage (LAA), with its rough trabeculae, plays a central role in the development of stroke during atrial fibrillation. The AtriClip^®^ (AtriCure, Mason, OH, USA) device for LAA exclusion offers a safe and effective approach to managing AF complications. This technique is typically performed in combination with other cardiac surgical procedures under direct or thoracoscopic visualization and under TEE guidance. However, during minimally invasive surgical procedures, spatial constraints within the surgical field may be a challenge when applying the AtriClip^®^ (AtriCure, Mason, OH, USA) [3,4,5]. Therefore, LAA occlusion is often performed through the transverse pericardial sinus during cardiac arrest. To improve TEE guidance for clip application, a beating heart technique has recently been described [5].

The IntraClude^®^ device can be used for the intraluminal occlusion of the aorta in patients undergoing a cardiopulmonary bypass with cardiac arrest. This device allows intraluminal pressure monitoring and the administration of cardioplegia during both conventional and minimally invasive cardiac surgery [6]. The inflated IntraClude^®^ balloon occludes the ascending aorta and must be applied carefully to avoid distal or proximal migration.

## 2. Case Presentation

**Figure 1:**
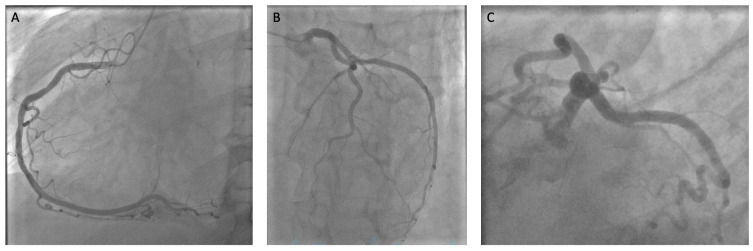
Preoperative coronary angiography excluding relevant stenosis. (**A**) View of Right Coronary Artery (RCA) without stenosis (LAO 30° CRA 0°) (**B**) View with irregularities in the Left Main Artery (LM) and smooth walls in Right Ventricular Artery (RIVA) and Right Circumflex Artery (RCx) (LAO 12° CRA 29°) (**C**) Spider View Showing no stenosis in the LM (LAO 41° CAU 26°).

A 62-year-old male patient (172 cm, 70.4 kg, BMI: 23.8 kg/m^2^) was referred to our university department of cardiac surgery with the diagnosis of symptomatic mitral valve regurgitation, tricuspid valve regurgitation and atrial fibrillation. The diagnosis included mild irrelevant aortic regurgitation, a patent foramen ovale, arterial hypertension and a combined pre- and post-capillary pulmonary hypertension. A therapy-relevant stenosing coronary artery disease was excluded by coronary angiography (Figure 1). The preoperative computed tomography of the aortic was performed to ensure the feasibility of performing a minimally invasive procedure and to exclude occlusive peripheral arterial disease.

Preoperative transesophageal echocardiography (TEE) showed severe mitral valve regurgitation with an eccentric regurgitant jet, a flail of the medial portion of the A3 segment, and a prolapse of the A3 segment. The vena contracta (VC) at 9–10 mm had an estimated effective regurgitant orifice (ERO) of 0.5 cm^2^ and an estimated regurgitation volume (RV) of 95 mL. Additionally, there was a prolapse in the septal tricuspid valve leaflet with severe tricuspid regurgitation, a VC of 9 mm × 10 mm, an estimated ERO of 0.4 cm^2^ and an estimated RV of 53 mL. The right ventricular ejection fraction was within the normal range.

## 3. Surgical Technique

Preoperatively, angio-CT confirmed the feasibility of a peripheral cardio-pulmonary bypass placement via the inguinal vessels with the cross-sectional diameter of the right common femoral artery measuring 14 mm. The ascending aorta had a diameter of 35 mm. Adhesions or anatomic contraindications for a minimally invasive procedure were excluded. A decision for surgical mitral and tricuspid valve repair was made in accordance with the 2021-ESC/EACTS guidelines for the management of valvular heart disease.

**Figure 2:**
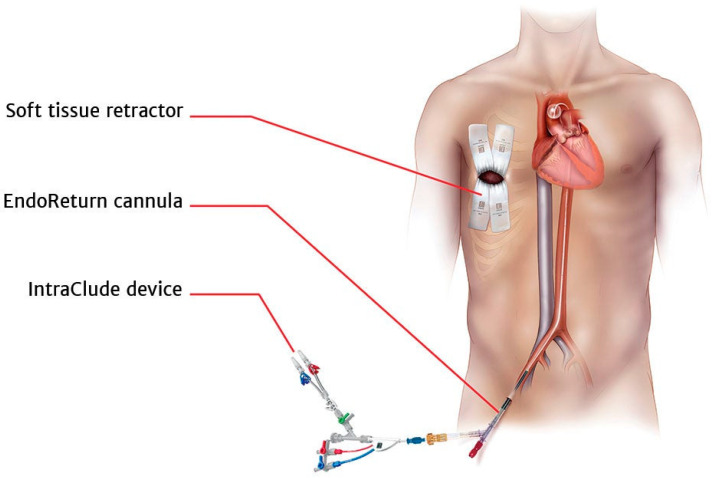
Depiction of the inflated Intraclude Device^®^ occluding the ascending aorta for LAAO during minimally invasive surgery. This device is well used for monitoring blood pressure in the aortic root as well as delivering cardioplegia (Courtesy of Edwards LifeScience, Nyon, Switzerland).

After a skin incision and preparing the right femoral artery and vein, the endoreturn arterial (ThruPort Edwards 23 mm) and bicaval venous peripheral cannulas (Estech RAP 23/25 Fr) were inserted and extracorporeal circulation was initiated. With the use of TEE guidance, the IntraClude^®^ balloon was positioned in the ascending aorta, with its tip located 20 to 30 mm distal to the aortic annulus at the sinotubular junction level, but was not inflated (Figure 2).

**Figure 3:**
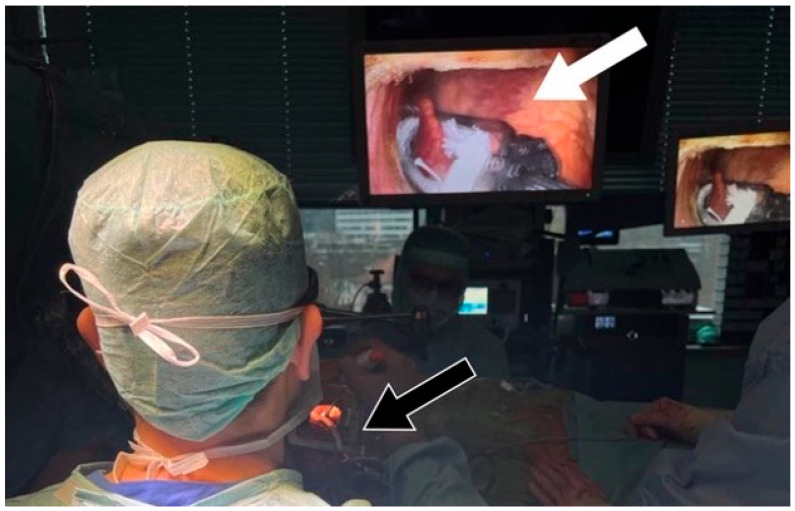
Intraoperative setting during the application of the AtriClip-Pro^®^ (AtriCure, Mason, OH, USA) for LAAO during minimally invasive surgery. Depicted is the lateral traction of the Jackson–Pratt drain (black arrow) exposing the LAA (white arrow).

A right mini thoracotomy was performed in the 5th intercostal space (ICS), and a 3D endoscopic camera (Aesculap “Eddy” 3D EinsteinVision Endoscope 30°) was inserted in the 3rd ICS. To enhance visualization, the diaphragm was fixed and pulled down using a U-suture. Subsequently, the right side of the pericardium was opened above the phrenic nerve, exposing the heart. A Jackson–Pratt drain was placed through the transverse pericardial sinus around the aorta, and with lateral traction, the LAA was exposed [5] (Figure 3).

**Figure 4:**
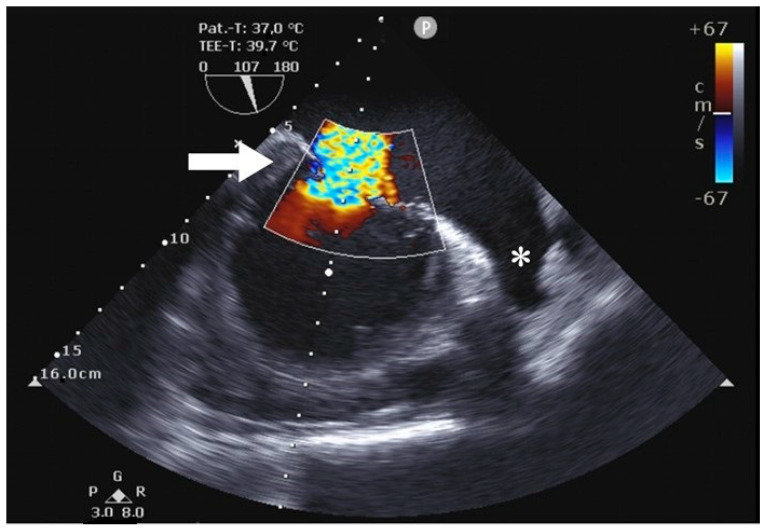
Preoperative transesophageal echocardiography showing severe mitral valve regurgitation (arrow) as well as the LAA (*).

**Figure 5:**
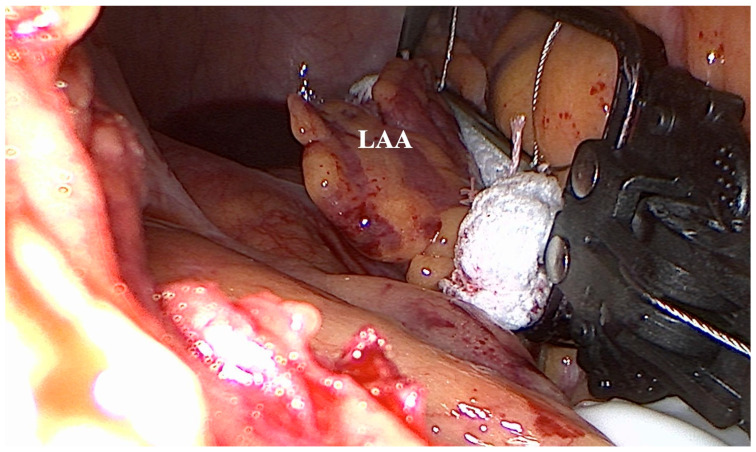
The AtriClip-Pro^®^ (AtriCure, Mason, OH, USA) device correctly placed at the base of the LAA before occlusion.

**Figure 6:**
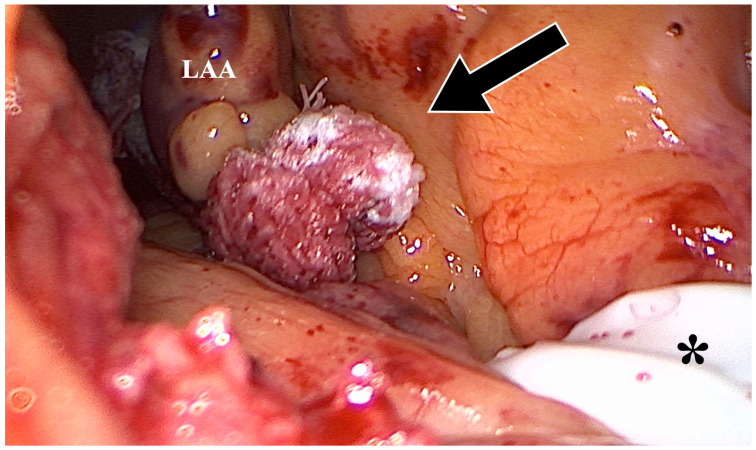
The AtriClip-Pro^®^ (AtriCure, Mason, OH, USA) (arrow) after its placement at the base of the LAA occluding it. (* exposition of LAA through the lateral traction using the Jackson–Pratt drain).

**Figure 7:**
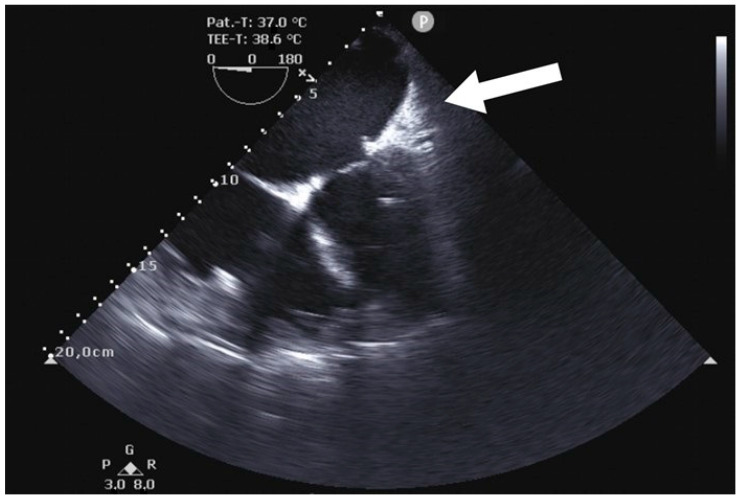
Postoperative transesophageal echocardiography showing the complete occlusion of LAA after correct placement of the AtriClip-Pro^®^ (AtriCure, Mason, OH, USA) device (P: Pointer on ultrasound transducer).

Using the AtriCure^®^ (AtriCure, Mason, OH, USA) measuring device, the LAA base size was measured. After bending the AtriClip-Pro^®^ (AtriCure, Mason, OH, USA) device, the clip was anteriorly passed over the LAA apex, gently compressing the great arteries and exposing the LAA. With a beating heart, the Atriclip-Pro^®^ (AtriCure, Mason, OH, USA) was adequately placed under TEE guidance (Figure 4 and Figure 5), and the device was released after the confirmation of LAA closure (Figure 6 and Figure 7; Supplementary Video S1).

**Figure 8:**
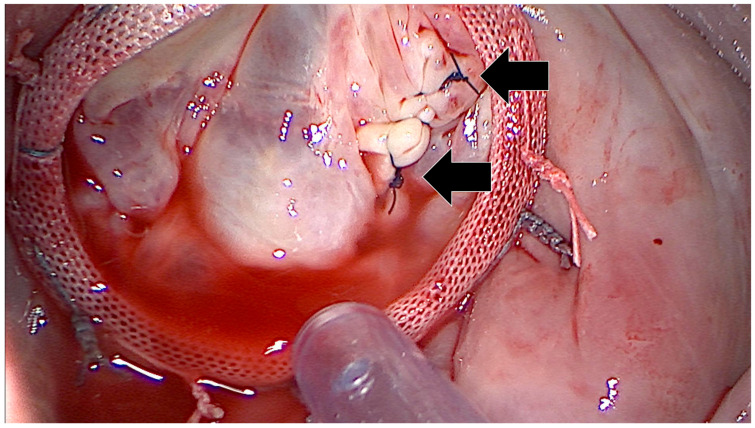
Mitral valve successfully reconstructed using commissural sutures (black arrows) and the implantation of an Edwards Physio II annuloplasty ring.

The interatrial sulcus was exposed by placing holding sutures. After the fibrillation of the heart, the ascending aorta was occluded with the IntraClude^®^ device and antegrade Brettschneider’s cardioplegia (overall volume: 1800 mL) was administered over 7 min. A transverse atriotomy was performed to expose the left atrium. Following a left-sided MAZE procedure, the mitral valve was successfully reconstructed using commissural sutures and the implantation of an Edwards Physio II annuloplasty ring (Figure 8). The visualized PFO could be closed by direct suture. The left atriotomy was closed after venting the left heart. After slinging both venae cavae, the right atrium was opened. The tricuspid valve was reconstructed with a 34 mm Edwards Physio 6200 Tricuspid Valve annuloplasty ring, and the right atriotomy was closed after venting the right heart. Upon deflating the aortic balloon, a sinus rhythm recurred. Intraoperative transesophageal echocardiography showed a good reconstructive result with a competent mitral and tricuspid valve and an effectively closed LAA. The complete postoperative course was uneventful, and the patient was discharged on postoperative day 9.

## 4. Discussion

To our knowledge, this is the first case in which LAA closure was performed on the beating heart under minimally invasive conditions using endoclamping.

Minimally invasive mitral valve procedures are becoming increasingly common. Small working ports make anatomy more difficult to visualize, and subsequently, the surgeon has less space to manipulate. In addition, the need to cross-clamp the aorta for cardiopulmonary bypasses increases these difficulties, as the surgeon has limited space to perform the aortic cross-clamping. As conventional aortic cross-clamping (exoclamping) is not always feasible, endoclamping techniques have been developed to improve a minimally invasive procedure approach [7].

The IntraClude^®^ device (Edwards Lifesciences) has been shown to be safe and to achieve results comparable with conventional exoclamping [8]. This makes endoclamping a viable alternative to conventional exoclamping without extending the clamping time or pump time, as well as in-hospital stay and postoperative outcome. However, endoclamping does have disadvantages, including a higher incidence of iatrogenic aortic dissections, challenges in aortic exposure, the instability of the endoclamp, as well as higher costs [8].

Different pharmacological, interventional and surgical options are available for the treatment of AF. The surgical closure of the LAA has become increasingly important in the mitigation of a stroke. In fact, stapler occlusion, including the resection of the LAA or the use of an epicardial device for LAA occlusion, such as the AtriClip^®^ (AtriCure, Mason, OH, USA) device, are common procedures. As other authors have shown [9], the mechanical occlusion of the LAA is not only safe and effective but also results in electrical isolation that reduces the risk of persistent or reoccurrence of AF by 28% [9].

The conventional approach for LAA occlusion typically involves performing it during cardiac arrest, which can unnecessarily prolong ischemia time. Additionally, this method limits the echocardiographer’s ability to assess occlusion efficacy using TEE due to cardiac emptying during ischemia. Consequently, our technique enables the real-time visualization of the occlusion performed by the surgeon. Furthermore, research has indicated that surgical LAA occlusion might not be as efficacious as the utilization of an external clip, such as the Atriclip^®^ (AtriCure, Mason, OH, USA). Given the thin and delicate nature of the atrial tissue, the early and delayed tearing of the surgical closure could occur, potentially leading to the reopening of the LAA orifice over time and the recanalization of the LAA [10] (Table 1).

The advantage of the “anterior pathway” for the application of an AtriClip^®^ (AtriCure, Mason, OH, USA) is the beating heart technique with TEE guidance, which is achieved by applying lateral traction to the aorta. Moreover, the use of a 30° endoscope provides a superior visibility of the structures posterior to the aorta, such as the LAA. This technique can also be applied when an IntraClude^®^ catheter has been inserted into the ascending aorta but should be performed before balloon inflation.

## 5. Conclusions

LAA occlusion is an effective surgical technique to prevent stroke from AF. As shown in the presented case, the use of AtriClip^®^ (AtriCure, Mason, OH, USA) during minimally invasive mitral valve replacement is possible, even if endoclamping is planned. However, if possible, the AtriClip^®^ (AtriCure, Mason, OH, USA) should be placed before the aortic occlusion with the IntraClude^®^ device.

## Figures and Tables

**Table 1:** Comparison between standard and minimally invasive operative approach.

	Sternotomy Plus Aortic Cross Clamping Supported MVR Plus LAA Ligation	Lateral Thoracotomy Plus Aortic Endo Clamping Supported MVR and LAA Clipping
Blood transfusion	More	Less
IntraClude^®^ Device	No advantage	More space for the surgeon to manipulate; less invasive with reduced skin incisions
Field of view for cardiac surgeon	Open	Restricted situs, but better visualization for mitral valve apparatus
LAA occlusion failure	Comparable if performed on beating heart and TEE-guided	
Reconvalence time	Longer due to sternotomy	Shorter
Morbidity and mortality	Comparable	
Cosmetics	Midchest big sternotomy scar	Small discreet scar

## Data Availability

Data sharing is not applicable to this article as no datasets were generated or analyzed during the current study.

## Supplementary Materials

The following are available online at https://www.mdpi.com/article/10.3390/jcm12196325/s1. Supplementary Video S1: Left Atrial Appendage Occlusion on a Beating Heart during Minimally Invasive Valve Surgery using Aortic Endoclamp.
